# Expected and Unexpected Effects of Pharmacological Antioxidants

**DOI:** 10.3390/ijms24119303

**Published:** 2023-05-26

**Authors:** Irina Tyuryaeva, Olga Lyublinskaya

**Affiliations:** Department of Intracellular Signaling and Transport, Institute of Cytology of the Russian Academy of Sciences, Tikhoretskii pr. 4, 194064 St. Petersburg, Russia; olga.lyublinskaya@incras.ru

**Keywords:** antioxidants, ROS, N-acetylcysteine, NAC, polyphenols, resveratrol, EGCG, curcumin, vitamin C, ascorbate

## Abstract

In this review, we have collected the existing data on the bioactivity of antioxidants (N-acetylcysteine, polyphenols, vitamin C) which are traditionally used in experimental biology and, in some cases, in the clinic. Presented data show that, despite the capacity of these substances to scavenge peroxides and free radicals in cell-free systems, their ability to exhibit these properties in vivo, upon pharmacological supplementation, has not been confirmed so far. Their cytoprotective activity is explained mainly by the ability not to suppress, but to activate multiple redox pathways, which causes biphasic hormetic responses and highly pleiotropic effects in cells. N-acetylcysteine, polyphenols, and vitamin C affect redox homeostasis by generating low-molecular-weight redox-active compounds (H_2_O_2_ or H_2_S), known for their ability to stimulate cellular endogenous antioxidant defense and promote cytoprotection at low concentrations but exert deleterious effects at high concentrations. Moreover, the activity of antioxidants strongly depends on the biological context and mode of their application. We show here that considering the biphasic and context-dependent response of cells on the pleiotropic action of antioxidants can help explain many of the conflicting results obtained in basic and applied research and build a more logical strategy for their use.

## 1. Introduction

The basis of life is a flow of electrons—from donor molecules, high-energy electrons pass to acceptor molecules in a chain of successive redox reactions. In the course of these reactions, energy stored in the form of ATP is produced and organic molecules which serve as metabolic fuels, such as carbohydrates or fatty acids, are decomposed. Aerobic organisms use oxygen as a final electron acceptor. Therefore, in the cascades of redox reactions, many active oxygen-containing compounds are formed, collectively termed as reactive oxygen species (ROS). Among these substances are non-radical (hydrogen peroxide H_2_O_2_, organic hydroperoxide ROOH, nitrogen-containing molecules, such as NO) and free radical (superoxide anion radical O_2_^−•^, hydroxyl radical OH^•^, peroxyl radical ROO^•^, etc.) species; a detailed list can be found in [1,2]. 

In 1956, Denham Harman put forward the theory that aging and age-associated diseases are the result of the damaging effects of free radicals [3]. In a later work, D. Harman proposed a way to prolong life by adding inhibitors of free radical reactions to the diet with a minimum intake of products that enter into such reactions [4]. D. Harman suggested that by postponing age-related diseases, the average life expectancy could thus be increased by 5 years or more, based on the average life expectancy at that time in countries with a developed health care system (approximately 70 years). A little more than 50 years have passed since then, and the role of ROS, both radical and non-radical, is recognized as important in both aging and the progression of many diseases, and the concept of inhibiting ROS production using supplemental antioxidants has become widespread in pharmacology. 

The term ‘antioxidants’ originally referred to substances that can inhibit oxidative processes in the production cycles of chemical and food industries. Fast progress in redox biochemistry in the second half of the 20th century led to the borrowing and subsequent spread of this term in life sciences. According to the biochemical definition formulated by Halliwell [1,5], an antioxidant is “any substance that delays, prevents, or removes oxidative damage to a target molecule”. Based on this definition, antioxidants can be understood as both endogenous molecules produced by cells, and exogenous supplements—substances of natural or synthetic origin. At the same time, recent advances in cell biology have proved that natural antioxidant protection is mainly performed in cells not by individual compounds, but by branched metabolic pathways consisting of many molecules with various functions. Therefore, at present, the term antioxidant, in the context of a single chemical compound, is usually understood as a pharmacological supplement.

The idea of using pharmacological antioxidants for health protection has been especially common in recent decades [6,7]. During this time, thousands of studies on the mechanisms of action and the effectiveness of various agents with antioxidant activity have been carried out both in research laboratories and in the clinic (682,032 documents in PubMed). Based on these studies, dietary recommendations have been developed with an emphasis on the use of plant foods rich in antioxidants, many new pharmacological antioxidants and protocols for the treatment of various pathologies using these medications have been developed, and antioxidant supplements have become an important part of vitamin complexes and cosmetics [8]. And, indeed, recent data show that the average life expectancy in 54 countries of the world has already exceeded 80 years (“The World Factbook—Central Intelligence Agency”, https://www.cia.gov/the-world-factbook/field/life-expectancy-at-birth/country-comparison, accessed on 19 March 2023). However, is this increase due to the use of antioxidants?

What benefits have been derived from the use of antioxidants, and are there any clinical successes due to the use of antioxidants? We turned to The Cochrane Library, a collection of evidence-based medicine databases containing independent, high-quality reviews based on more than 1.8 million clinical trials. The keyword “antioxidant” (search in Title, Abstract, Keyword) found 72 Cochrane Reviews summarizing the results of dozens of clinical trials of antioxidants in healthy people and people with various diseases, and 6 Clinical Answers—practical clinical recommendations for the use of antioxidants formulated considering the evidence-based results.

According to expert opinions, pharmacological antioxidants show very modest success in clinical practice, and, moreover, can even be harmful in certain conditions. A positive effect of antioxidant supplementation has been noted for the reproductive health of men aged 20 to 52 years with oligospermia or asthenospermia, but the evidence is of low certainty and does not allow definitive conclusions [9]. There is a positive effect of the use of antioxidants in women with polycystic ovary syndrome—low-certainty evidence suggests that antioxidants may increase the frequency of pregnancy in subfertile women and increase live births [10]. Antioxidant multivitamin and mineral supplements may slow the progression of age-related macular degeneration (AMD), neovascular AMD, and vision loss in adults with AMD [11]. Other beneficial effects from the use of antioxidants have not been proven. At the same time, it was found that overall mortality (healthy people and people with various diseases) was on average slightly higher in the group taking antioxidant supplements (by 0.3%, beta-carotene, vitamin E and vitamin A), which seems to be all significant when considering that experts analyzed a very large amount of evidence from 78 randomized clinical trials involving 296,707 patients [12].

Antioxidant supplementation can cause side effects such as hemorrhagic stroke (vitamin E), nosebleeds (vitamin E; low-quality evidence), skin rashes (multivitamins; moderate-quality evidence), and stimulate the progression of lung cancer (vitamin C in women, vitamin A in smokers and asbestos exposures, β-carotene; high-quality evidence) [13,14]. Most reviews of clinical trials with antioxidants conclude that the level of evidence for antioxidant efficacy and absence of side effects is insufficient to unambiguously assess an effect from the use of antioxidants, and that more well-designed randomized placebo-controlled trials are needed to keep hope for the prospect of using antioxidants in clinical practice.

What is the reason for such uncertainty with the use of antioxidants? Have not enough experiments been conducted establishing that antioxidants are able to protect cells in vitro and in vivo from damage and death under the oxidative stress conditions? In recent years, the scientific literature has accumulated a sufficient amount of experimental data to answer these questions. All the accumulated evidence can be briefly summarized as follows: based on the definition of Halliwell, antioxidants represent a wide class of biologically active substances that are absolutely different in their mechanisms of actions, and which are characterized by the following features:

- Antioxidants entail a wide range of molecular events in cells that are difficult to predict in advance, since their redox interactions affect an extensive network of reactions related to all aspects of cell physiology (further referred to as pleiotropic activity, an ability of biologically active substances to cause certain biological (pharmacological) effect by implementing more than one mechanism [15]);
- Antioxidants are able to cause bidirectional hormetic responses at the level of both individual cell and whole organisms (biphasic effect, stimulation and inhibition of any form of biologic activity, depending on their dose [16]);
- Antioxidants act differently on cells and tissues in different biological contexts, and experiments on cell culture or even animal models rarely fully reproduce the effects that antioxidants cause in the human body (context-dependent behavior).

In this review, we consider antioxidant compounds that are most commonly exploited in experimental biology, and are also used as drugs, supplements, or part of the human diet. Systematization of the existing experimental data in terms of the features listed above proves that this approach help to explain many contradictory results obtained in fundamental and applied research on the biological activity of these substances. In addition, using these compounds as an example, we demonstrate that pharmacologically active antioxidants have the ability not to suppress, but stimulate the redox processes in cells. Interestingly, they are able to activate both aerobic and hydrosulfide metabolisms, further proving that sulfur- and oxygen-mediated reactions cooperatively form a complex network of metabolic and regulatory redox pathways in human organism.

## 2. N-acetylcysteine

**Figure 1:**
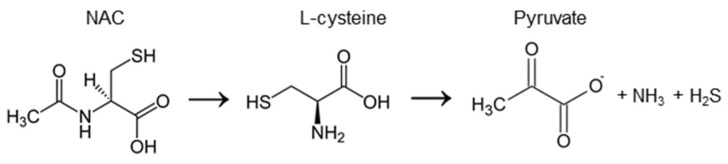
Chemical structure of NAC as well as one of the pathways of its enzymatic conversion that results in H_2_S generation. For more details, see [18].

N-acetylcysteine (NAC), also referred to as N-acetyl-L-cysteine, is a cysteine prodrug (Figure 1), which is included on the World Health Organization’s list of essential medicines [17]. In therapy, NAC is used as a mucolytic agent and as an antidote against paracetamol overdose. Fundamental studies often explain these and many other health-promoting and cytoprotective effects of NAC by its direct ability to scavenge oxidants and reduce oxidized proteins. Actually, this agent is among the most widely used antioxidants in experimental biology. Indeed, many experiments on cell cultures and animal models proved that NAC is able to protect cells and tissues from oxidative damage; however, recent works show that the real molecular mechanisms of its protective action have yet to be established [18].

In humans, the absolute bioavailability of oral NAC is low and varies between 6–10%, probably due to rapid metabolism in the gut [19]. It was reported, that in healthy subjects, oral application of 600 mg of NAC leads to the 2.6–2.7 mg/L (~15 μM) plasma concentrations [19]. At the same time, after NAC intravenous infusions, serum concentration can achieve 300 mg/L (~1.8 mM at the loading dose of 125 mg/kg^−1^h^−1^ for 15 min) [20]. NAC is negatively charged at physiological pH and its passive transport across the cell membranes is hampered—the level of NAC was found to be 5-fold lower in human red blood cells than that in plasma after the intravenous infusions [20]. NAC can be converted to cysteine either extracellularly, or intracellularly, after its uptake by cells. NAC transporter has not been univocally identified so far (hypothetically, it is AE1, Anion Exchanger 1 [21]), in contrast to its derivative cysteine, which is neutral in its reduced form and can be transported by ASCT1 (Alanine/Serine/Cysteine/Threonine Transporter 1 also known as Neutral Amino Acid Transporter A [22]).

### 2.1. The Chemical Activity of NAC

In vitro research identified several mechanisms as potential causes of the cytoprotective and regulatory effects of NAC on cells (the comprehensive review can be found in [18]). First, similar to other small monothiols, NAC has the ability to scavenge ROS and reduce disulfide bonds in proteins or other sulfur-containing molecules [23,24]. However, low reactivity and correspondingly slow kinetics limit these mechanisms to situations where NAC concentration is high—within the millimole or even mole range [25]. Accordingly, in cell culture experiments, a direct antioxidant effect of NAC can theoretically be expected, at least in the extracellular space, however, it can hardly be assumed in vivo. Secondly, NAC is considered as a source of cysteine for the biosynthesis of glutathione (GSH), a critical cofactor for many antioxidant pathways. Both in vitro and in vivo evidence exists for NAC-mediated stimulation of GSH biosynthesis, at least under conditions when GSH is depleted due to some GSH consuming impacts on cells, for example paracetamol poisoning [26]. Finally, probably the most physiologically relevant pathway of intracellular conversion of NAC is associated with hydrogen sulfide (H_2_S) generation. NAC-produced cysteine can be enzymatically desulfurated, which causes H_2_S generation (Figure 1) and subsequent formation of sulfane sulfur species—per- and polysulfides (RSSH and RSSnSR, respectively) which exert multiple metabolic and regulatory functions in cells [27]. 

**Figure 2:**
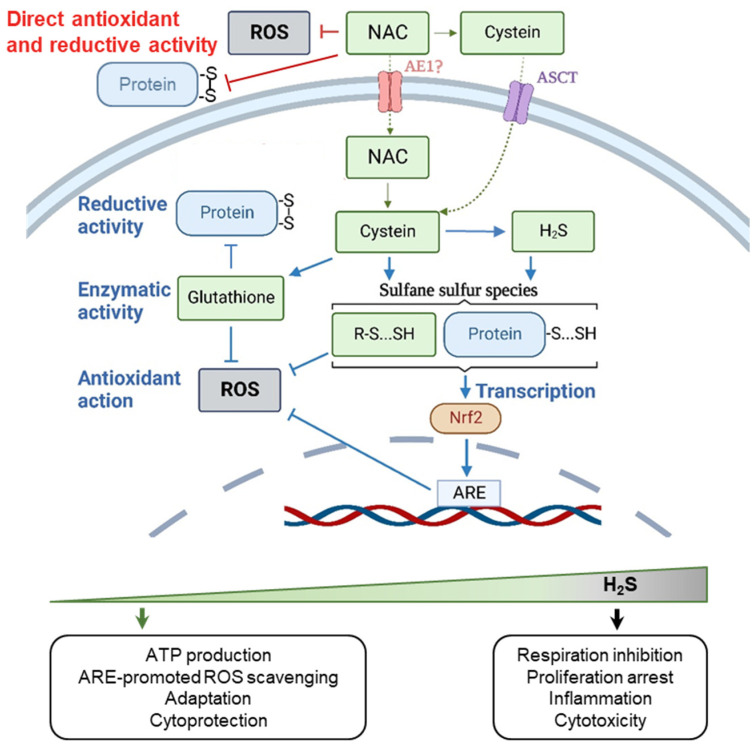
Biological activity and hormetic effects of NAC. Intracellular antioxidative activities of NAC are provided by NAC-derived metabolites—GSH and H_2_S. NAC is able to stimulate the biosynthesis of glutathione under conditions of its depletion in cells but can be ineffective in elevating GSH levels under normal conditions. Generation of H_2_S can cause a wide set of biphasic hormetic responses. Blue arrows mark effects which NAC can cause both in vitro and in vivo. Red arrows mark processes which can be realized under certain conditions in cell culture experiments but which have not been proved to occur in vivo (with only one exception—when NAC is applied at high, non-pharmacological concentrations directly to the lesion); generally, these effects are difficult to expect in vivo due to insufficient achievable concentrations of NAC and/or kinetic rate constants of corresponding reactions. Abbreviations: AE1, Anion exchanger 1; ASCT1, Alanine/Serine/Cysteine/Threonine Transporter 1; Nrf2, nuclear factor erythroid 2-related factor 2; ARE, antioxidant/electrophile response element, GSH, glutathione (created with BioRender.com).

H_2_S-mediated pathways are related to the primordial anaerobic metabolism, to sulfur-dependent bioenergetics, and hence are highly abundant and conservative [28]. Hydropersulfide groups (-SSH) can act as a direct radical scavengers, cap the thiol groups of cysteine residues, protecting them from oxidative damage, regulate the activity of the signaling proteins to trigger stress-adaptive responses [29,30] (Figure 2). For instance, it has been shown [31] that H_2_S-mediated persulfidation reactions can cause the activation of Nrf2 (nuclear factor erythroid 2-related factor 2) transcription factor that plays a key role in maintaining cellular homeostasis, especially under oxidative stress conditions. Its activity is subject to multilevel regulation, but the main redox-dependent negative regulator of Nrf2 is the Kelch-like ECH-associated protein 1 (Keap-1), which directs Nrf2 to the proteasomal degradation under normal reducing conditions [32]. Upon oxidation or alkylation of Keap-1 thiol groups, Nrf2 ubiquitination is interrupted, which leads to its stabilization in the cytoplasm, regulated transport to the nucleus, and regulated binding to the antioxidant/electrophile response element (ARE/EpRE). The latter is located in the promoter regions of genes coding antioxidant proteins, detoxification enzymes, and xenobiotic transporters that provide protection against oxidative stress, inflammation, senescence, and apoptosis. The H_2_S-mediated formation of Cys151-persulfide (Cys151-SSH) in the Keap-1 can also cause its dissociation from Nrf2, leading to Nrf2 translocation into the nucleus and triggering the transcription of protective genes [31]. Thus, it is highly plausible that, along with persulfide-assisted scavenging of ROS, the antioxidant effects of NAC in cell culture and animal models can also be explained by the H_2_S-mediated Nrf2 activation. Recent evidence demonstrated that the membrane permeable NAC modification (N-acetylcysteine amide) provided neuroprotection and attenuated oxidative stress in rats following traumatic brain injury via the activation of the Nrf2-ARE signaling pathway [33]. 

Finally, it has been recently proved that NAC can interact also with the phospholipids of cell membranes—it can alter the hydration bilayer in the polar head of the lipid membrane and cause changes in the dynamics of the membrane, which may partly explain its mucolytic effects, because phospholipids present in the pulmonary surfactants promote mucociliary clearance [34].

### 2.2. Biphasic Effect of NAC

Since NAC is considered one of the most proven agents that protect cells from oxidative stress, a huge amount of in vitro research using NAC has been conducted aimed at developing approaches to treat various pathologies associated with increased ROS production. Among them was those focused on the immunomodulatory activity of NAC and its ability to influence proliferation and activation status of T cells. Surprisingly, some of these studies indicated that NAC has an inhibitory effect on T-cell-mediated immunity and activation of the transcription factor NF-kB [35,36,37], while others have suggested an immunopotentiating effect [38,39]. In 2011 the study by Karlsson et al. proved that NAC may have dual and opposing effects on the T cells, depending on the dose [40]. Low concentrations of NAC (<3 mM) substantially upregulated the expression of lymphocyte activation markers (CD25 and CD71) and increased the proliferation of activated T cells, and high concentrations of NAC (>10 mM) exerted the opposite effects. 

In addition to T cells, research on other cell types also proved the biphasic effect of NAC on cell proliferation—in 2005, using two human carcinoma lines and primary human epidermal keratinocytes, it was found that low NAC doses (<0.5 mM) potentiate cell proliferation, whereas millimole range of concentrations had an inhibitory effect [41]. In recent years, a lot of studies on cell culture models have been published showing that modulation of cell redox status induced by high doses of NAC (typically exceeding 5 mM) disturbs cell cycle regulation and blocks cell division [42,43,44,45,46]. NAC applied at millimolar concentrations was shown to affect the level/activity of the cell cycle regulators needed for DNA replication—it decreased the levels of cyclins D1 [42] and A2 [47], increased the activity of APC/C (Anaphase-Promoting Complex/Cyclosome), an ubiquitin E3-ligase, which controls the level of mitotic cyclins, including cyclin A2 [47], suppressed the activity of CDK2 (Cyclin-Dependent Kinase 2) which ensures the initiation of DNA replication [46]. The relationship between these effects has not yet been established, but two studies [42,46] coupled NAC-mediated disturbances in the cell cycle regulation to the alterations in the level of ROS produced by mitochondria and/or changes in the mitochondrial redox metabolism. 

Interestingly, the causes of NAC biphasic activity have not been identified. However, generally, when considering NAC as a precursor of H_2_S, the biphasic responses of cells to NAC treatments do not seem surprising, since the biphasic and hormetic effects of H_2_S are well known (Figure 2) [28]. For instance, dual and opposing effects on both cell proliferation and mitochondrial redox metabolism were well documented [48]. 

### 2.3. Context-Dependent Behavior of NAC

Despite the wide palette of effects that NAC can potentially cause in biological systems, which scenario is realized in cells under conditions in vitro, and even more so in vivo, is largely determined by the specific circumstances of NAC application. First, as we discussed above, much depends on the final concentration of the drug in cells, which, in case of in vivo applications, may be affected by their localization in the body and on the method of drug administration. For example, when NAC is applied as mucolytic, direct exposition of sputum to NAC at high-millimolar and molar concentration in vitro or in experimental animals with pulmonary pathology (using aerosols or lung infusions), its ability to reduce disulfides and to have a thinning effect on sputum has been reliably proven [49,50]. At the same time, in the case of oral use of the drug, which leads to completely different concentrations in the lung tissues, the ability of NAC to correct pulmonary pathologies due to its direct reductive activity is questionable and, apparently, is explained by completely different effects [51,52]. 

In addition to the method of drug administration, the activity of NAC in biological systems is also determined by the physiological context of its use. For example, under conditions of total glutathione depletion (for example in reactions with xenobiotics, including paracetamol) NAC is effective in replenishing GSH [26], which contributes indeed to NAC cytoprotective effects in vitro and in vivo, but, under normal conditions, it can be ineffective in elevating GSH levels [53,54,55]. Moreover, accumulating data obtained in cell culture experiments prove that, having a cytoprotective effect under conditions of oxidative stress, under normal physiological conditions, millimolar concentrations of NAC can themselves serve as a stressful factor, causing different responses in various cellular compartments. For example, applications of NAC at 4 mM concentration to both human and hamster cells have been shown to trigger rapid oxidation of the redox-sensitive biosensor Grx1-roGFP2 in the mitochondrial matrix, but did not challenge the redox environment in the cell cytoplasm [56]. Importantly, the similar effect has been previously observed in vivo—feeding NAC to fruit flies induced oxidation of mitochondrial, but not cytosolic, roGFP2-based probes expressed in various tissues [57]. Dr. Ezeriņa and colleagues [27] clearly showed that this effect is due to the NAC-induced generation of H_2_S. Catabolism of H_2_S is thought to occur primarily via intramitochondrial oxidation, which may result in the enhanced pro-oxidative tone inside mitochondria at high-dose NAC treatments. It is known that H_2_S can act as both an antioxidant and a pro-oxidant in biological systems, stimulating the formation of disulfide bonds between the vicinal thiols of proteins, including roGFP2 [18,27,58,59]. In addition, it has been shown that in cultured human mesenchymal stem cells, 10 mM NAC stimulates a transcriptional response, indicating the induction of endoplasmic reticulum stress [47]. The molecular mechanisms underlying this phenomenon can also be related to the H_2_S-mediated disturbances in the thiol-disulfide metabolism that cause inappropriate folding of proteins in the endoplasmic reticulum, but this is yet to be established. 

In vivo, the context-dependent bioactivity of NAC was most clearly manifested when testing this agent as an antitumor drug. Whilst multiple evidence exist that NAC treatment inhibits proliferation of cancer cells from different tumors in cell culture experiments [60], it occurred to be ineffective and even dangerous in treating tumors (or at least some kinds of them) in vivo. For example, dietary NAC supplementation markedly increased tumor progression and decreased survival in mouse models of B-RAF- and K-RAS-induced lung cancer by suppressing ROS and DNA damage, increasing cell proliferation in tumors and downregulating p53 expression, whose antitumor activity is known to be stimulated by ROS and DNA damage [61]. Importantly, parallel experiments in cell cultures showed that NAC increased the proliferation of human lung cancer cells with wild-type, but not mutant, p53 [61]. In addition, another study [62] has shown that pretreatment of melanoma cells with NAC prior to their intravenous administration to mice enhances tumor cell migration and metastasis via RhoA activation and increased glutathione synthesis. These observations indicate that the context-dependent pleiotropic activity of NAC and NAC-produced metabolites should be taken into account when developing protocols for its clinical applications.

To conclude, recent evidence suggests that disulfide-reducing, antioxidative and cytoprotective activities of NAC in cellulo are facilitated mostly by NAC-derived metabolites (GSH, H_2_S, per- and polysulfides) rather than its direct antioxidant action. Further study will help to elucidate how these metabolites contribute to some paradoxical effects of NAC in biological systems, including biphasic impact on cell proliferation, cell cycle dysregulation and ER stress induction observed at millimole NAC concentrations in cell culture experiments, as well as intriguing results regarding the tumor-potentiating activity of NAC in vivo.

## 3. Polyphenols: Resveratrol, Curcumin, Epigallocatechin Gallate

**Figure 3:**
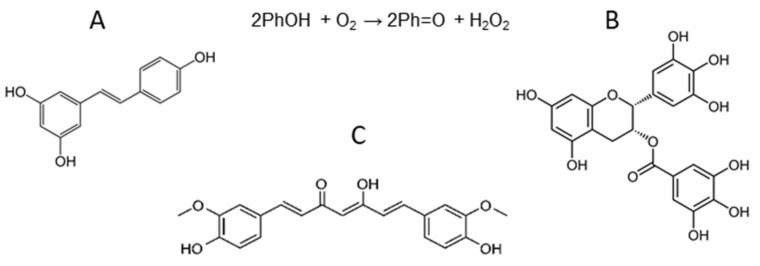
Chemical structure of resveratrol (**A**), epigallocatechin gallate (**B**), and curcumin (**C**), as well as the reaction of PPh auto-oxidation which results in H_2_O_2_ generation. Abbreviations: PhOH, reduced polyphenols; Ph=O, polyphenol quinone.

Polyphenols (PPhs, in the singular—PPh) are secondary metabolites of plants bearing aromatic rings with one or more hydroxyl groups (Figure 3) making them strong chemical antioxidants. PPhs are produced in plants enzymatically from the phenylalanine amino acid and can be divided into non-flavonoids (such as resveratrol, sesamin, and ellagic acid), and flavonoids (such as curcumin, epigallocatechin gallate, apigenin, and quercetin). Due to their natural origin, these substances are often considered safer than conventional pharmaceutical drugs and regarded now as beneficial nutritional supplements. 

In vivo, most PPhs are characterized by low-to-mid bioavailability, which is attributed to their instability, poor absorption within the gastrointestinal tract and rapid metabolism [63]. Multiple studies have indicated that the half-life and plasma concentrations of PPhs’ metabolites are significantly higher than that of the native compounds after their oral administration [64,65]. The metabolism of PPhs is specific to different organs and tissues of the body, since it depends on pH, concentration of PPhs themselves, oxygenation and availability of catalysts—metals and enzymes participating in their chemical conversion. Moreover, differences in pharmacokinetics caused by the different activity of enzymes catalyzing PPh transformations have been shown also between various species (mice, rats and humans) [66]. That is why animal studies do not allow full extrapolation of a safe dose, efficacy, and modes of PPh application to humans.

The concentration of PPhs found in human plasma after their oral administration varies between substances. In the case of epigallocatechin gallate (EGCG), it can reach up to 1 μM after a single dose of EGCG-containing extracts [66]. At the same time, only trace levels of curcumin and resveratrol were found in the peripheral circulation after their oral uptake [64,67]. However, the PPh concentration in tissues may be higher than that in circulation—apparently these agents can accumulate in different organs due to their ability to bind with proteins and phospholipids in the plasma membranes of cells. Cellular uptake of PPhs occurs mainly by passive transport, but their intracellular amount is regulated by different efflux pumps, which also limits their bioavailability [65].

### 3.1. The Chemical Activity of Resveratrol, Curcumin, and Epigallocatechin Gallate

Resveratrol is found in many plant species and is highly concentrated in the skin of red grapes. It consists of two phenolic rings bonded together by a double styrene bond, which forms the 3,5,4′-trihydroxystilbene (Figure 3A). The chemical structure of resveratrol leads to low water solubility (<0.05 mg/mL). In contrast, EGCG, the most abundant and active tea catechin, is soluble in water at least to 5 mg/mL. EGCG consists of two phenolic rings (A and B rings), bearing 3 hydroxyl groups at the B ring, and dihydropyran heterocycle (C ring) with a gallate moiety esterified at carbon 3 (Figure 3B). Curcumin is a phenolic compound extracted from *Curcuma longa* rhizome commonly used in Asia as a spice and pigment. Curcumin is a crystalline powder practically insoluble in water. Its molecule consists of two aromatic ring systems containing o-methoxy phenolic groups, connected by a seven carbon linker consisting of an α,β-unsaturated β-diketone moiety (Figure 3C). 

The bioactivity of PPhs is highly pleiotropic. On the one hand, O-H bonds of polyphenolic hydroxyl groups have low energy of dissociation due to a combination of electronic and steric effects, which makes PPhs active reductants and free radical scavengers. The direct antioxidant activity of PPhs was observed in the gastrointestinal tract of both experimental animals and humans, where taken orally PPhs were shown to prevent lipid peroxidation and generation of advanced glycation and lipid oxidation end-products [68]. At the same time, instability and low absorption by enterocytes, which cause low concentration of PPhs in blood and peripheral tissues, do not allow PPhs to be considered competitive antioxidants outside the gastrointestinal tract. Instead, it occurred that PPhs can affect the redox environment in tissues by exerting not antioxidant but pro-oxidant effects. In the presence of oxygen, PPhs can be oxidized, or even auto-oxidized, generating PPh semiquinone radicals (Ph-O^•^) and PPh quinone (Ph=O) [69,70,71]. As a byproduct of this reactions H_2_O_2_ is formed (see Figure 3). PPh pro-oxidant action has been proven both in vitro and in vivo and depends on the presence of metal ions, environment oxygenation, pH and PPh concentration [72]. 

**Figure 4:**
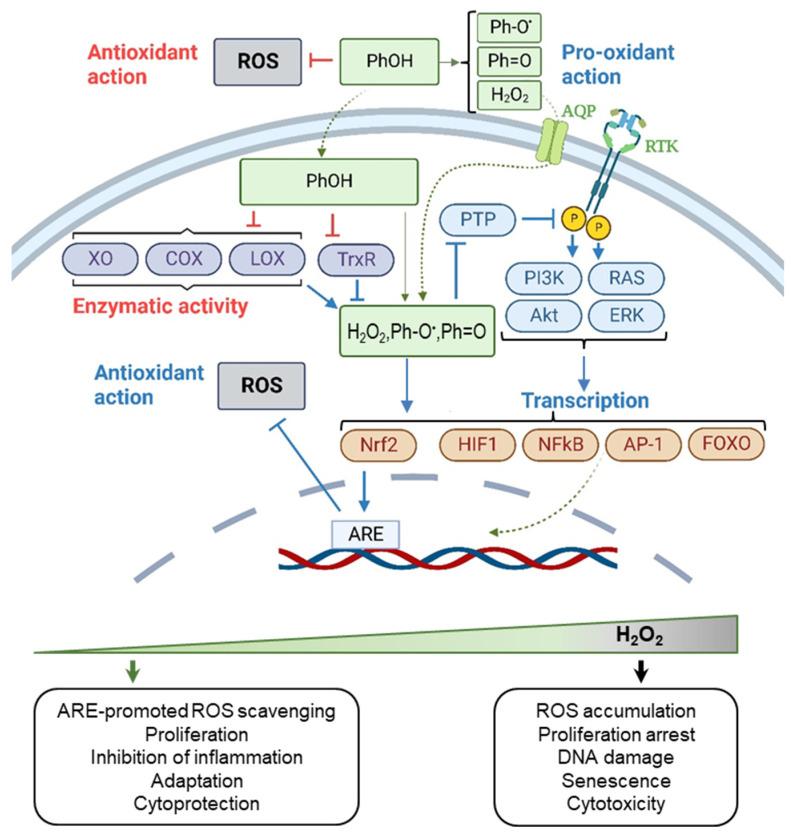
Biological activity and hormetic effects of PPhs. Intracellular activity of PPhs is mainly explained by their capacity to produce pro-oxidants (H_2_O_2_, Ph-O^•^, Ph=O) which cause various hormetic effects in cells. For instance, H_2_O_2_, when produced at low concentrations, stimulates multiple signaling events and promotes Nrf2 activation, which is now considered as a main cause of the antioxidant and cytoprotective effect of PPhs both in cell culture and animal model studies. In contrast, at high concentrations, H_2_O_2_ can produce harmful effects. Blue arrows mark effects which PPhs can cause both in vitro and in vivo. Red arrows mark processes that can be realized under certain conditions in cell culture experiments, but which have not been proved to occur in vivo (with only one exception—inside GIT after the PPhs’ oral uptake); generally, these effects are difficult to expect in vivo due to insufficient achievable concentrations of PPhs and/or kinetic rate constants of corresponding reactions. Abbreviations: PhOH, reduced polyphenols; Ph-O^•^, PPh semiquinone radical; Ph=O, PPh quinone; AQP, aquaporin; RTK, receptor tyrosine kinase; PTP, protein tyrosine phosphatase; PI3K, phosphoinositide 3-kinase; Akt, protein kinase B; RAS, Ras GTPase; ERK, extracellular signal-regulated kinase; Nrf2, nuclear factor erythroid 2-related factor 2; ARE, antioxidant/electrophile response element; HIF1, hypoxia-inducible factor 1; NFkB, nuclear factor kappa B; AP-1, activator protein 1; FOXO, Forkhead box O; XO, xanthine oxidase; COX, cyclooxygenase; LOX, lipoxygenase; TrxR, thioredoxin reductase (created with BioRender.com).

PPh-induced formation of pro-oxidants (H_2_O_2_, Ph-O^•^, Ph=O) leads to the activation of different signaling pathways in cells (Figure 4). For example, it is the ability of H_2_O_2_ to inhibit phosphatases (e.g., PTP1B) that may facilitate the PPh-induced activation of PI3K-Akt and RAS-ERK1/2 pathways [73,74,75,76,77,78], as well as their down-stream targets—transcription factors HIF1, NF-kB1, AP-1, FOXO1, and Nrf2 (reviewed in [65]), which stimulate cytoprotection. In addition, pro-oxidants generated by PPhs may directly cause Keap-1 modification [16] and Nrf2-specific cellular gene expression reprogramming that activate the endogenous antioxidant defense (described in the Section 2.1). Importantly, Nrf2 activation stimulated by PPhs has been proved both in vitro and in vivo. For example, it was shown that resveratrol in combination with another PPh, quercetin, can activate the Keap-1/Nrf2 antioxidant defense in rats with metabolic syndrome, thus protecting them against oxidative stress and causing health-promoting effects [79]. Resveratrol can also act as an indirect antioxidant via the Nrf2 signaling pathway in the brain tissue of Alzheimer’s disease mouse model and improve the spatial abilities of animals [80]. 

In addition to the pro-oxidant activity of PPhs, these compounds have the ability to indirectly affect the intracellular redox homeostasis, at least under conditions in vitro, by chelating the metals (iron and copper), which influences Fenton chemistry [81], and by inhibiting some redox-active enzymes (e.g., thioredoxin reductase [82,83], xanthine oxidase [84,85,86], cyclooxygenase and lipoxygenase [87]). Finally, what is also important, PPhs and their metabolites can produce effects not associated with the cellular redox metabolism—they can form complexes with human serum albumin and lipoproteins, as well as interact with receptor proteins and phospholipids in the cell membranes [88,89,90,91,92]. All these types of chemical activity can affect functioning of PPhs in vivo, influencing their therapeutic potential.

### 3.2. Biphasic Effect of Resveratrol, Curcumin, and Epigallocatechin Gallate

In general, PPhs can generate cytoprotective and beneficial effects or, on the contrary, can induce cell damage. For example, multiple studies in vitro showed that in many cases PPhs demonstrate antiapoptotic properties when apoptosis is induced by certain chemicals or mutagens. Importantly, these effects were observed both in case of normal and cancer human cells [93]. In unstressed cells, typically, an increased cellular fitness is observed at extracellular concentrations up to several dozens of micromoles, whereas higher concentrations tend to produce toxic effects, leading to a hormetic dose–viability curve. For example, an increase in the cell viability was observed at resveratrol concentrations less than 50 µM in a set of different human cell lines, while higher concentrations led to a concentration-dependent toxicity [94]. Studies of human mesenchymal stem cells showed that 0.1 µM resveratrol promoted self-renewal, whereas concentrations above 5 µM inhibited self-renewal and increased senescence rate [95]. The molecular mechanisms underlying resveratrol-stimulated senescence induction in cultivated mesenchymal stem cells have been studied in [96]. It was found that high concentrations of drug (>20 μM) caused replication stress which was accompanied with DNA strand breaks accumulation and cell senescence induction. Hormetic responses of different types of cultured cells to EGCG and curcumin are comprehensively reviewed in [97] and [98], respectively.

Hormetic responses to PPhs were well documented not only in vitro but also in vivo. For example, long-term (30 days) administration of resveratrol to a group of rats at three different dozes (2.5 mg/kg, 25 mg/kg and 100 mg/kg) was shown to provide cardioprotection at lower doses and detrimental effects at higher doses [99]. Similarly, in a mouse model of indomethacin-induced gastric ulcers, resveratrol at a dose of 2 mg/kg caused an accelerated recovery, but at a dose of 10 mg/kg delayed healing of ulcers [100]. Finally, while low-dose resveratrol administration (25 mg/kg) partly improved renal function in mice with kidney damage caused by unilateral ureteral obstruction, the high-dose administration (50 mg/kg) aggravates renal fibrosis instead [101]. Although in humans PPhs are generally well tolerated, some adverse effects, including nephrotoxicity and gastrointestinal problems, were reported as well [102,103]. For instance, whilst 450 mg/day of resveratrol was reported to be a safe dose for a 60 kg person [104], the dosage of 1000 mg/day or above was reported to inhibit cytochrome P450 isoenzymes [105] and to elevate biomarkers of CVD risk in overweight older adults [106].

Discussing the potential mechanisms of the hormetic responses of cells to PPhs, recent studies show that many of the dose-dependent effects, both in vitro and in vivo, are explained not by the dual capacity of PPhs to directly suppress and stimulate ROS production, but exclusively by their pro-oxidant effects [16,65] (Figure 4). PPh-induced oxidative activation of the Nrf2-ARE pathway leads to an increased activity of endogenous antioxidant systems of cells, which, apparently, is of key importance for the realization of the antiapoptotic and cytoprotective properties of PPhs, especially when these substances are used as antistress agents [107]. At the same time, at high concentrations of PPhs, the oxidative load on cells due to their pro-oxidant activity may exceed the adaptive capacity of cells and cause cytotoxicity.

### 3.3. Context-Dependent Behavior of Resveratrol, Curcumin, and Epigallocatechin Gallate

Due to the specific pharmacokinetics and variety of chemical transformations of PPhs occurring in vivo, the response of cells and organisms to the treatments with these substances is always ambiguous and is largely determined by the physiological context. For example, in [72], it has been shown that acute resveratrol administration can exert opposite effects on tissue lipoperoxidation in rats depending on the time of drug administration—resveratrol behaves as a pro-oxidant during day time and as an antioxidant at night. In experimental models of different pathologies, administration of PPhs also shows variable effects: positive, negative, or completely neutral [108]. It has been shown that in animals, resveratrol treatment can improve cardiovascular function by reducing myocardial ischemia-reperfusion injury, vasodilation and atherosclerosis [109], has several neuroprotective roles in various neurodegenerative impairments, such as Alzheimer′s, Huntington′s and Parkinson′s diseases, amyotrophic lateral sclerosis and alcohol-induced neurodegenerative disorders [110], and can prevent inflammation [111]. However, at the same time, it can act as a thyroid disruptor and a goitrogen [112]. Multi-directional activity of PPhs is proposed to be due to varying gut microbiota, bioavailability and pharmacokinetics of PPhs, which may depend on the physiological context in case of various pathologies.

Cochrane Reviews summarizing the results of hundreds of clinical trials of dietary/supplemental PPhs in healthy volunteers or people with various diseases conclude that research is insufficient to evaluate the safety and efficacy of resveratrol supplementation for treatment of adults with type 2 diabetes mellitus, and that there is insufficient and conflicting evidence regarding the effect of dietary uptake of PPhs on the risk of colorectal neoplasms (flavonoids) as well as on the overall risk of cancer (green tea extract) [113,114,115].

To conclude, PPhs are characterized by low bioavailability, rapid metabolism and complicated pharmacokinetics in vivo. Yet, despite this, they show a relevant biological efficacy in animal models of inflammatory, cardiovascular, and neurodegenerative impairments. Therapeutic effects of PPhs are often explained by their ability to affect signaling and transcription. This ability is promoted mainly by PPh metabolites, especially pro-oxidative metabolites, including H_2_O_2_. Pro-oxidant action, which depends on the physiological context and concentration of PPhs, results in the Nrf2 activation, which is now considered as a main cause of the antioxidant and cytoprotective effect of PPhs both in cell culture and animal model studies. Generally, administration of PPhs may cause pleiotropic and hormetic effects in cell cultures, experimental animals and humans, and animal studies do not allow full extrapolation of PPh efficacy to humans. Safety and efficacy of PPhs need to be further investigated when considering their pharmacologic applications.

## 4. Vitamin C 

Vitamin C (ascorbic acid, ascorbate) is a vital organic compound that is not synthesized in humans, unlike most mammals, and must be provided with food as an essential dietary component. The clinical manifestation of vitamin C deficiency is scurvy, which is expressed by fatigue, myalgia, divergence of wounds, loosening and loss of teeth, hemorrhages, and leading to death without treatment. Symptoms indicate connective tissue defects, lack of noradrenaline and, possibly, other hormones. 

**Figure 5:**
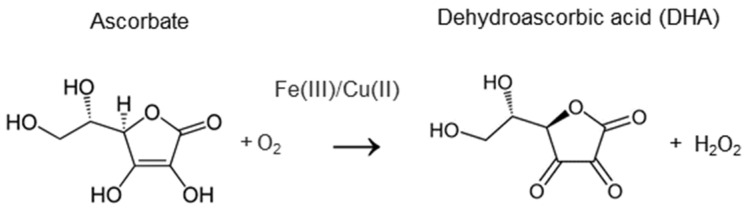
Chemical structure of vitamin C as well as metal-catalyzed reaction of ascorbate oxidation which results in H_2_O_2_ generation.

The pharmacokinetics of vitamin C is complicated [116]. It exists in the body in three forms—ascorbate (reduced form), free radical of ascorbate (relatively long-lived and low active), which is formed after the donation of one electron, and dehydroascorbic acid (DHA, an oxidized product) arising after the transfer of the second electron—in the bloodstream it contains approximately 5% [117] (Figure 5). Ascorbate enters cells via SVCTs (sodium-dependent vitamin C transporters) 1 and 2; SVCT2 is found in all tissues of the human body, and SVCT1 is found in the intestine, kidney tubules, liver, skin and lungs, and is characterized by several nucleotide polymorphisms. DHA is transported into cells by several glucose transporters (GLUTs) and most cells recycle DHA to ascorbate [118,119]. When taken orally, ascorbate is adsorbed from the intestine through SVCT1 into the bloodstream in a concentration-dependent manner: at a dose of up to 200 mg, the bioavailability of ascorbate is 100%, but with an increase in the dose, the bioavailability decreases and at a dose of 1.25 g it is approximately 50% [120]. Plasma concentrations of ascorbate are normally 40–65 µM and have a saturation limit of approximately 80 µM when drug is taken orally [120]. Ascorbate is distributed differentially between tissues and organs, and accumulates in tissues at much higher concentrations than in plasma due to the SVCT-assisted transport. The highest level of ascorbate is detected in the brain and adrenal glands (up to 10 mM), several times less was found in the liver and lungs, and several hundred micromoles—in muscles, heart and kidneys [118]. The distribution of ascorbate in the body is tightly controlled, mainly due to the different expression of SVCT2 in cells and tissues, as well as retention mechanisms existed in some organs, such as the brain. It is important to note that the recommended daily intake of ascorbate increased from 10 mg to 90–100 mg in recent years. At such dose, plasma ascorbate concentration is of approximately 50 μM, and further increase in the daily dose has no advantage [119]. At the same time, given that the estimated daily turnover of ascorbate in healthy non-smokers is only 3%, to replenish ascorbate, a healthy diet rich in fresh vegetables and fruits seems to be quite enough [121].

### 4.1. The Chemical Activity of Vitamin C

In redox chemistry, ascorbate acts as an electron donor, thus being a potent reducing agent and free radical scavenger [122]. It can neutralize O_2_^−•^, OH^•^, O_3_, NO_2_^•^, as well as ROO^•^ [1], and is often used in chemical research aimed at the development of new antioxidant agents as a positive control—to assess the antioxidant potency of newly synthesized substances [123,124,125]. Consistent with this, studies on cell cultures showed that ascorbate loaded to cells in the form of DHA can prevent massive DNA damage and mutations caused by H_2_O_2_-innduced oxidative stress [126]. However, how important the role of supplemental ascorbate in counteracting the oxidative stresses in the body, in competition with endogenous extra- and intracellular antioxidant systems, is still under debates [119,122]. From the one hand, experimental studies proved the effect of ascorbate supplementation on the prevention of oxidative DNA damage in humans after exposure to xenobiotics provoking the oxidative stress [127], as well as on the certain types of oxidative protein damage in subjects with low basal level of ascorbate [128]. However, on the other hand, intervention studies have not proved a reduction in biomarkers of oxidation or clinical benefit when using ascorbate for the prevention and treatment of pathologies associated with oxidative imbalances [122,129]. The latter may be explained, at least partly, by the saturable absorption of ascorbate by humans. In contrast, the ability of pharmacologic concentrations of ascorbate to induce oxidative stress [130] is well documented both in vitro and in vivo. It has been shown that ascorbate can generate H_2_O_2_ in the oxidation reactions catalyzed by iron or copper [131] (Figure 5) and that H_2_O_2_ was detected in extracellular fluid (but not blood) of experimental animals after the intravenous administration of ascorbate [132]. Due to its ability to generate H_2_O_2_, ascorbate can cause activation of the Nrf2-ARE pathway (described in Section 2.1), which was confirmed by the experiments on both cell cultures and animal models [133,134]. 

**Figure 6:**
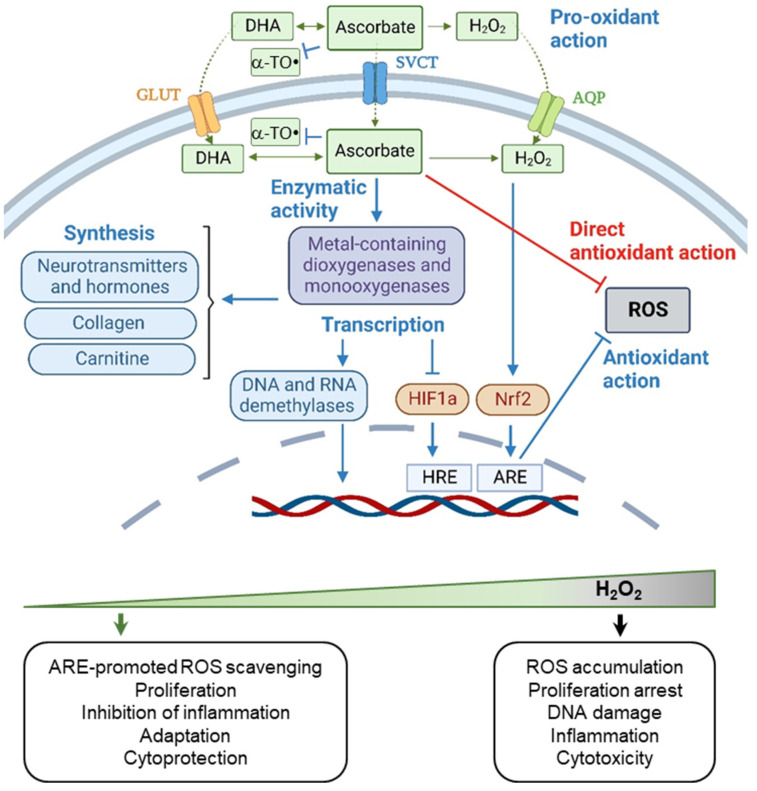
Biological activity and hormetic effects of ascorbate. Antioxidant action of ascorbate both in cultured cells and in vivo is influenced by its ability to modulate cell transcriptional responses, in particular, by activating/suppressing the activity of Nrf2 and HIF-1a transcription factors and by modifying the epigenetic status of DNA. Pro-oxidant action of ascorbate, arising from the ability to generate H_2_O_2_ in the presence of metal ions, promotes the hormetic response of cells to ascorbate supplementation. Blue arrows mark effects which ascorbate can cause both in vitro and in vivo. Red arrows mark processes which have been observed in cell culture experiments but which have not been univocally proved by clinical trials on humans. Abbreviations: α-TO•, α-tocopheroxyl radical; DHA, dehydroascorbate; SVCT, sodium-dependent vitamin C transporter; GLUT, glucose transporter; AQP, aquaporin; Nrf2, nuclear factor erythroid 2-related factor 2; ARE, antioxidant/electrophile response element; HIF1, hypoxia-inducible factor 1; HRE, hypoxia response element (created with BioRender.com).

The most physiologically relevant and well-studied redox function of ascorbate delivered to human body with food is its reducing activity (Figure 6). Ascorbate is known to recover α-tocopherol (vitamin E), the first-line antioxidant neutralizing the lipid peroxyl radicals which forms as a result of lipid oxidation [135]. Accordingly, dietary vitamins C and E act synergistically as potent antioxidants protecting the cell membranes from the oxidative damage. In addition, ascorbate participates as an electron donor in the intracellular enzymatic reactions as a cofactor and cosubstrate for iron-containing dioxygenases involved in collagen synthesis [136], carnitine formation [137], regulation of translation and gene expression [138,139,140], as well as for two copper-containing monooxygenases involved in the synthesis of hormones by neurons and endocrine tissues [141]. Intriguingly, the exact mechanism of ascorbate action in these reactions has not been established, but it is evidently associated with the maintenance of metals in a reduced state in the active center of enzymes. 

By influencing the activity of dioxygenases, ascorbate is able to regulate adaptive cell responses to hypoxia and other metabolic stresses through the regulation of the stability and activity of oxygen-dependent transcriptional regulator HIF-1a (hypoxia-Inducible Factor 1-alpha) [142] (Figure 6). Ascorbate deficiency can stabilize HIF-1a by inhibiting the enzymatic reaction of HIF-1a proline hydroxylation and contributes thus to the accumulation of HIF-1a, its translocation to the nucleus, and triggering HIF-induced expression of target genes in the absence of hypoxia. In addition, ascorbate deficiency can block the factor inhibiting HIF (FIH), which hydroxylates HIF-1a asparagine, also leading to the aberrant HIF activation [138]. On the contrary, accumulation of ascorbate can stimulate HIF-1a degradation under hypoxia [142]. Among the other dioxygenases regulated by ascorbate are also erasers of epigenetic modifications, such as JHDMs (Jumonji-C domain-containing Histone Demethylases) and the TET (ten-eleven translocation) family of DNA hydroxylases. Correspondingly, numerous studies have revealed that addition of ascorbate to the culture medium of somatic cells during reprogramming stimulates epigenetic rearrangements and improves the efficiency and quality of induced pluripotent stem cell formation [143,144,145]. 

### 4.2. Biphasic Effect of Vitamin C

When used as a supplement, ascorbate is able to activate the biphasic hormetic response in cells [16]. Its cytoprotective effects, usually observed at low concentrations, is often facilitated by Nrf2-mediated transcription of antioxidant genes (Figure 6). For instance, ascorbate has been shown to activate the Nrf2-ARE pathway in rat RAW 264.7 macrophages both at basal and LPS-induced stress conditions when present at micromole (0.01–0.3 mM) concentrations, suppressing the pro-inflammatory reaction of macrophages to LPS (bacterial endotoxin) [133]. In line with that, experiments in vivo showed that ascorbate reduced inflammation and increased the survival of endotoxemic mice [133]. On the other hand, addition of higher (0.3–20 mM) doses of ascorbate to cultured cells is known to cause extracellular H_2_O_2_ production in the amounts that lie beyond the cytoprotective range, which is now considered to be perspective for treating cancer [146]. However, recent studies show that the biphasic response of cancer cells to ascorbate treatments is also possible and should be taken into account when considering ascorbate as antitumor drug. For instance, in colorectal cancer cell lines with weak SVCT-2 expression, high-dose L-ascorbic acid (>1 mM) showed anticancer effects but low-dose (<10 μM) treatment induced cell proliferation by upregulating cyclin D1 and c-Myc [147]. 

### 4.3. Context-Dependent Behavior of Vitamin C

The biological activity of supplemental ascorbate strongly depends on the context. For example, its intracellular accumulation is determined by the number of SVCT channels on the cell membrane, and its ability to generate H_2_O_2_ is affected by the presence of metal ions. In addition, its effect on cells depends on the redox context and the ability of ascorbate to act in synergy with other modulators of cell redox metabolism in case of their co-supplementation. For instance, experiments on cell cultures have shown that ascorbate, due to its pro-oxidant action, can facilitate the activation of the Nrf2-ARE pathway [133]; however, at the same time, it can counteract the induction of Nrf2-ARE signaling caused by the administration of resveratrol to cells [134]; high concentrations of ascorbic acid increase H_2_O_2_ production in cell culture medium, providing a stress signal that disrupts the Nrf2 signaling [134]. Another striking example—studies in animals addressed whether ascorbate protects against lipid peroxidation. Ascorbate appeared to be protective when it was administered before the oxidative stress agent paraquat, and at the same time, the ascorbate accelerated oxidative stress when it was administered after paraquat, by interacting with metal ions released from damaged cells [148]. Finally, there is still considerable controversy regarding the role of ascorbate in preventing oxidative DNA damage in humans, since this protective effect strictly depends on the presence of free iron and the basal level of vitamin C in the body [149]. For example, oral supplementation with vitamin C in human volunteers decreased H_2_O_2_-induced DNA damage in isolated lymphocytes but had no effect on endogenous levels of DNA damage [150]; however, when healthy subjects received co-supplements of iron and ascorbate, the levels of oxidative DNA damage in white blood cells were elevated after 6 weeks of supplementation [151]. Importantly, the latter was observed only in the group of volunteers with a high initial level of plasma vitamin C (approximately 70 μM), while in a second group of volunteers with a lower mean level (approximately 50 μM), presupplemental levels of oxidative DNA damage were higher and decreased on supplementation with iron and ascorbate. 

Currently, ascorbate is being actively tested as an antitumor drug. In vitro research have shown that high (millimole) doses of ascorbate, which generate extracellular H_2_O_2_ at concentrations ≥25 µM, are cytotoxic to various tumor but not normal human cells [146,152]. In animal models, it has been shown that, after intravenous administration of ascorbate, H_2_O_2_ is detected in extracellular fluid [132], the excretion of ascorbate from the tumor is significantly prolonged, and the pro-oxidant damaging effect on cancer cells is reinforced by the suppression of the transcription factor HIF-1 [153]. Finally, clinical trials proved that millimolar (10–20 mM) concentrations of ascorbate in the blood and extracellular fluid, achieved by intravenous infusion (10 g/day for approximately 10 days and then orally), significantly increased the survival of patients with end-stage cancer [154,155]. Thus, clinical study of the use of high doses of vitamin C in the treatment of cancer patients, especially in combination with chemo-, radio- or immunotherapy, is now experiencing a second wave of interest, and is considered as a very promising and non-toxic method of cancer treatment [156]. However, at the same time, taking into account the multidirectional effect of HIF-1 and Nrf2 activity on carcinogenesis depending on the stage of the disease and the type of tumor, further fundamental and clinical studies considering the context-dependent behavior of ascorbate are obviously needed. Importantly, it has been shown not so far that therapy with a high dose of ascorbate can be risky for patients with some types of cancer treatments; ascorbate at the dose of 40 mg/kg/day orally was found to significantly reduce the activity of bortezomib treatment in vivo in the human multiple myeloma xenograph model [157]. Another striking stimulus to study the context-dependent behavior of ascorbate is the findings of the Cochrane Review [14], summarizing results of three randomized controlled trials, which concluded that in women, but not in men, ascorbate increased the risk of lung cancer incidence (high-certainty evidence).

In sum, ascorbate is characterized by specific pharmacokinetics, which is determined by its chemical lability, dose-dependent saturable absorption and distribution via active transport. Due to the capacity of various tissues in human organism to accumulate significant amounts of ascorbate, the body’s ascorbate requirements may well be met by dietary interventions. Similar to NAC and polyphenols discussed above, biological effects of supplemental ascorbate both in cultured cells and in vivo appeared to be determined to a greater extent by its ability to modulate cell transcriptional responses, in particular, by activating/suppressing the activity of HIF-1a and Nrf2 transcription factors and by influencing the epigenetic status of DNA. How important the role of supplemental ascorbate as a direct antioxidant in humans is still under debates. In contrast, its pro-oxidant action, arising from the ability to generate H_2_O_2_ in the presence of metal ions, is well documented now both in vitro and in vivo and considered to be perspective for treating cancer. Taking into account the dual, both inhibiting and promoting, role of HIF-1a and Nrf2 transcription factors in cancer pathogenesis, as well as pleiotropic and context-dependent behavior of ascorbate, further research is needed to develop the strategies of its potential clinical use.

## 5. Discussion

**Figure 7:**
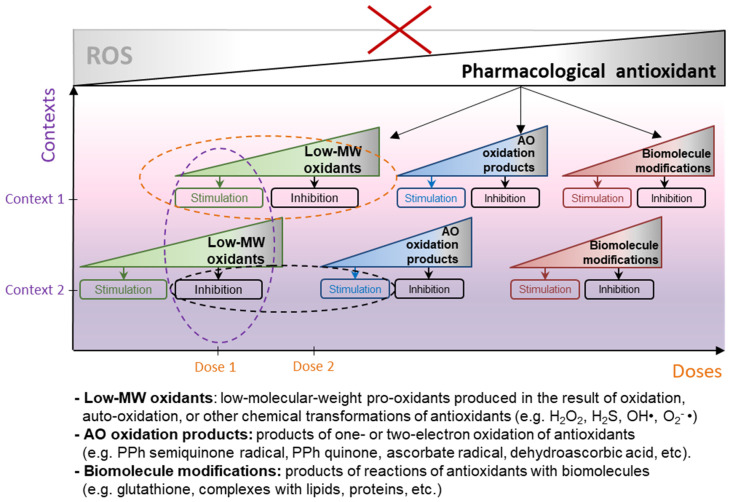
Pleiotropic action of pharmacological antioxidants: product-dose-context plot. In vivo, the ability of pharmacological antioxidants (those considered in this review and many others) to suppress ROS metabolism has not been proved. There is no unequivocal relationship between the concentration of antioxidants and intracellular level of ROS—neither in experiments on cell cultures, nor in animal models, much less in clinical trials on humans. The scheme shows how these drugs really act in biological systems—they produce low-molecular-weight pro-oxidants, pro-oxidative metabolites, interact with biomolecules (amino acids, lipids, proteins, etc.), thus activating intracellular redox metabolism. The overall outcome strictly depends on the dose and biological context. The opposite effects of pharmacological antioxidants (stimulation and inhibition of cell proliferation, adaptation, inflammatory reactions, DNA damage, cellular senescence, etc.) do not contradict each other but are explained by the dose- and context-dependent mode of their pleiotropic action.

The mechanisms of action of pharmacological antioxidants, or what are commonly referred to as ‘antioxidants’, are being clarified, highlighting the complex network of chemical reactions and signaling pathways. Recent data prove that biomedical definition of antioxidants needs to be revised. The direct ability of most supplemental antioxidants to scavenge ROS in vivo and in cellulo has been shown to be kinetically inconsistent, due to the low rates of corresponding reactions and/or their low bioavailability [16]. The idea of modulating redox homeostasis of cells using exogenous nucleophiles turns out to be a failure—the cellular molecular machines occurred to be much more active in ROS scavenging. Evolution, constantly ensuring the adaptation of living systems to changing conditions of oxygenation, has protected them from oxidative damage by developing extremely effective enzymatic pathways that control intracellular redox processes. Moreover, the redox metabolism of cells still retains traces of evolutionary changes—conservative metabolic pathways dating back to the times when oxygen was not readily available to living cells, and the electron flows necessary for energy production proceeded due to the redox reactions mediated by hydrosulfide [28]. Pharmacological antioxidants, which we consider in this review, have the ability to activate the redox processes of both aerobic and hydrosulfide metabolisms. And it is this ability to stimulate, rather than suppress, as previously thought, the redox activity of cells that explains the cytoprotective properties of antioxidants. Actually, supplemental antioxidants can be classified as ‘redox-activators’. Many of them can produce low-molecular-weight pro-oxidants (such as H_2_O_2_ or H_2_S) which cause oxidation of protein sensor thiols and facilitate hormetic reaction of cells to mild stress. In this case, the antioxidant action of drugs is mimicked by the Nrf2-ARE pathway, which controls the transcriptional response to oxidative stress and xenobiotics in cells. Having, fortunately, limited bioavailability, supplemental antioxidants keep the Nrf2 system in a healthy tone, and maintain so-called nucleophilic tone [16] in cells, granting them increased resistance to oxidative stress. However, at high concentrations of the drugs, when the adaptive response of cells to their pro-oxidant action is depleted, ‘antioxidants’ become toxic. Thus, in most cases, the effect that supplemental antioxidants exert on human cells and tissues is bi-phasic, and antioxidants are able to both stimulate and inhibit cellular activity, depending on their dose (see Figure 7).

Due to the capacity of supplemental antioxidants to stimulate intracellular redox metabolism, they affect the activity of various transcription modulators and/or cellular enzymes. Therefore, their activity is highly pleiotropic and accompanied by the context-dependent reaction of cells to these drugs. These circumstances complicate developing the strategies of antioxidants’ clinical applications for the treatment of pathologies accompanied by impaired redox homeostasis (cardiovascular diseases, inflammatory conditions, neurodegeneration). Importantly, this is also a serious obstacle for antioxidant therapy of cancer. Multiple evidence exist that Nrf2, which is targeted by antioxidants, can not only prevent the development of cancer, but vice versa, potentiate the development of cancer and provide resistance to antitumor therapy, depending on the stage of disease and type of tumor [158,159]. Depending on the specific conditions, it may be necessary not to activate, but, on the contrary, to inhibit Nrf2 to treat cancer [113,114,115].

## 6. Conclusion Remarks

Summarization of reviewed here studies allows us to re-consider the experimental results on pharmacological antioxidants, which previously seemed mutually exclusive (see Figure 7). The dual effects of antioxidants on cell proliferation, adaptation, senescence, immunomodulation, carcinogenesis, etc., seem quite natural if we take into account three parameters (product-dose-context) that govern the bioactivity of the drugs. Considering these features will help to better build the logic of experiments that use antioxidants and thereby avoid unfounded conclusions, as well as to design correctly the clinical trials aimed at the use of antioxidants in therapy. Overall, the benefits from antioxidant therapy should be substantiated by further research.

## 7. Future Prospects

Despite a critical re-evaluation of the prospects for the clinical use of antioxidants, the search for new ways to modulate the redox homeostasis in human body is constantly going on. Indeed, a large number of pathologies is accompanied and complicated by the aberrant course of extra- and intracellular redox reactions. In addition, to study redox metabolism and regulation, researchers need tools to target ROS produced by cells. However, to search for such opportunities, it is obviously necessary to develop new, more systemic approaches. These approaches may include (1) the development of principles for the classification of existing and newly synthesized antioxidants based on the mechanisms of their action; (2) the development of a database of existing antioxidants linking their structure and function; (3) the elaboration of tools (including those based on the use of artificial intelligence) to predict the structure of antioxidants with predefined properties; (4) the implementation of chemical computational methods and artificial intelligence for predicting the molecular interactions of newly developed substances and the set of pathologies that can potentially be corrected with their use; (5) a detailed experimental studies of the molecular interactions of each potential antioxidant and a thorough verification of the proposed effects in preclinical and clinical trials; (6) the rejection of the concept of indiscriminate suppression of ROS at the level of the whole organism and focusing on the targeted effects at the level of individual tissues, cell types, cellular compartments and/or individual chains of redox reactions.

What are the current trends in antioxidant research that might form a basis for the implementation of new strategies?

- An example of possible classification of substances with antioxidant activity is given in the recent review [6], where antioxidants are divided into: (1) capable of directly interacting with ROS and ensuring their elimination (for example, mimetics of antioxidant enzymes); (2) substances that activate the endogenous system of antioxidant defense in cells (for example, Nrf2 activators); (3) agents that reduce the harmful effects of oxidative stress (for example, iron chelators that prevent DNA damage, or sulfur-containing substances that prevent protein hyper-oxidation). Implementation of such classification will help to determine the set of pathologies that can potentially be corrected with the use of each type of antioxidants.
- Another direction of hot topical studies is the ongoing search for ways of targeted modulation of redox processes in cells and tissues. These studies have a very different focus, and a striking example is the development of mitochondria-targeted antioxidants. This work began back in the 1970s, when it was first proposed to use lipophilic tri-phenylphosphonium (TPP) cations to deliver various substances into mitochondria [160,161]. Since then, many mitochondria-targeted antioxidants have been synthesized—MitoVitE (TPP-linked vitamin E [162]) MitoQ (TPP-linked ubiquinone [163]), mitoTEMPOL (TPP-linked piperidine nitroxide [164]), and the series of SkQ antioxidants (TPP-linked plastoquinone-based compounds [165]). A more recent line of research is a development of liposome-encapsulated antioxidants and mitochondria-penetrating peptides (reviewed in [7]). SkQ1 has shown efficacy in phase 2 clinical trials for dry eye syndrome [166], and clinical trials of MitoQ for restoring kidney and cardiac function are ongoing (NCT03960073, NCT03586414).

## Data Availability

Not applicable.
